# Identification of a Biomarker in Cerebrospinal Fluid for Neuronopathic Forms of Gaucher Disease

**DOI:** 10.1371/journal.pone.0120194

**Published:** 2015-03-16

**Authors:** Hila Zigdon, Alon Savidor, Yishai Levin, Anna Meshcheriakova, Raphael Schiffmann, Anthony H. Futerman

**Affiliations:** 1 Department of Biological Chemistry, Weizmann Institute of Science, Rehovot, Israel; 2 de Botton Institute for Protein Profiling, The Nancy and Stephen Grand Israel National Center for Personalized Medicine, Weizmann Institute of Science, Rehovot, Israel; 3 Institute of Metabolic Disease, Baylor Research Institute, Dallas, TX, United States of America; Nathan Kline Institute and New York University School of Medicine, UNITED STATES

## Abstract

Gaucher disease, a recessive inherited metabolic disorder caused by defects in the gene encoding glucosylceramidase (GlcCerase), can be divided into three subtypes according to the appearance of symptoms associated with central nervous system involvement. We now identify a protein, glycoprotein non-metastatic B (GPNMB), that acts as an authentic marker of brain pathology in neurological forms of Gaucher disease. Using three independent techniques, including quantitative global proteomic analysis of cerebrospinal fluid (CSF) in samples from Gaucher disease patients that display neurological symptoms, we demonstrate a correlation between the severity of symptoms and GPNMB levels. Moreover, GPNMB levels in the CSF correlate with disease severity in a mouse model of Gaucher disease. GPNMB was also elevated in brain samples from patients with type 2 and 3 Gaucher disease. Our data suggest that GPNMB can be used as a marker to quantify neuropathology in Gaucher disease patients and as a marker of treatment efficacy once suitable treatments towards the neurological symptoms of Gaucher disease become available.

## Introduction

Gaucher disease (GD), the most common lysosomal storage disease (LSD), is caused by mutations in the *GBA1* gene, which encodes for glucosylceramidase (GlcCerase), the lysosomal hydrolase responsible for glucosylceramide (GlcCer) degradation [1]. GD is classically divided into three clinical sub-types based on age of onset and on signs of nervous system involvement [2]. Type 1 is the chronic, non-neuronopathic form and types 2 and 3 are the acute and chronic neuronopathic forms, respectively, which display central nervous system (CNS) involvement in addition to systemic disease [3], and are collectively known as neuronopathic GD (nGD). However, the disease encompasses a wide spectrum of phenotypes and a great diversity in severity and symptoms is observed in patients classified as the same sub-type. Thus, the manifestation of disease can be described as a phenotypic continuum. An effective treatment, enzyme replacement therapy, is available for type 1 GD but no therapies are available for nGD, although attempts are being made to identify possible therapeutic targets [4,5]. However, because of the wide heterogeneity of symptoms displayed by nGD patients, the efficacy of candidate drugs would be immensely facilitated by the availability of genuine biochemical biomarkers. Moreover, interest in GD and nGD has recently been boosted by the realization that heterozygous mutations in *GBA1* are a major risk factor for Parkinson’s disease [6], leading to the suggestion that GD therapies might be of use for treating Parkinson’s disease [7].

In the current study, we performed liquid chromatography/tandem mass spectrometry (LC-MS/MS) quantitative proteomics to identify biochemical markers in the cerebrospinal fluid (CSF) of four type 3 GD patients and five controls, and identified a protein, glycoprotein non-metastatic B (GPNMB), whose levels in the CSF reflect diseases severity. This was confirmed in a series of studies in which GD was induced in mice and GPNMB levels monitored in the CSF. We suggest that GPNMB can be used as an authentic biochemical marker to follow the progression of nGD pathology and the efficacy of potential treatments.

## Materials and Methods

### Human brain and CSF samples

The spinal fluid samples were collected for biomarker discovery from a clinical trial “a phase I/II randomized, controlled study of OGT 918 in patients with neuronopathic GD” (Clinicaltrials.gov identifier NCT00041535) [8]. The samples were collected under a study that was overseen by the Institutional Review Board (IRB) of the National Institute of Neurological Disorders and Stroke (NINDS), National Institute of Health (NIH). All patients or their legal guardians gave their written informed consent for their participation. Following a waiver of consent received from NINDS IRB, these samples became part of the Repository Protocol Institute of Metabolic Disease that is overseen by the IRB of Baylor Research Institute, Dallas, Texas. The stated purpose of this study was to “To support the neurometabolic research using in human samples and data in the Institute for Metabolic Diseases, Baylor Research Institute, Baylor University Medical Center”. Samples were anonymised prior to shipment. All patients were on long-term enzyme replacement therapy (ERT) as well as on Miglustat; note that neither have any therapeutic effect on the brain [8] All patients eye movement abnormalities [9] and had not undergone splenectomy. Human brains were provided by the University of Miami Brain and Tissue Bank for Developmental Disorders through NICHD contract NO1-HD-8–3284 [10]. All control brains were frozen within 6–26 h of death. GD patients were classified before death as types 1, 2 or 3 based on the clinical course of the disease, and in most cases, mutational analysis was also performed. Brains from GD patients were obtained post-mortem with informed consent between 7 and 22 h after death. After removal, brains were frozen on dry ice.

### Mouse tissues

Mice were maintained under specific pathogen-free conditions and handled according to protocols approved by the Weizmann Institute Animal Care Committee according to international guidelines. Gba^flox/flox^; nestin-Cre mice were used as a model of nGD, in which GlcCerase deficiency is restricted to neurons and macroglia [11,12]. nGD was also induced in C57BL/6OlaHsd mice by intra-peritoneal injection with 100 mg/kg/day conduritol B-epoxide (CBE) (Calbiochem), an irreversible GlCerase inhibitor [13].

### LC-MS/MS

Proteins were reduced by incubation with 5 mM dithiothreitol (Sigma-Aldrich) for 30 min at 60°C followed by alkylation with 10 mM iodoacetamide (Sigma-Aldrich) in the dark for 30 min at 21°C. Proteins were subsequently digested with trypsin (Promega) overnight for 6 h followed by trypsin for 16 h at 37°C. Digestions were stopped by addition of trifluroacetic acid (1%, v/v). Samples were stored at -80°C.

ULC/MS grade solvents were used for all chromatographic steps. Samples were loaded using split-less nano-Ultra Performance Liquid Chromatography (10 kpsi NanoAcquity, Waters). The mobile phase was (A) H_2_O + 0.1% (v/v) formic acid and (B) acetonitrile + 0.1% (v/v) formic acid. Desalting of the samples was performed online using a reverse-phase C18 trapping column (180 μm internal diameter, 20 mm length, 5 μm particle size; Waters). Peptides were separated using a C18 T3 HSS nano-column (75 μm internal diameter, 250 mm length, 1.8 μm particle size; Waters) at 0.25 μl/min. Peptides were eluted from the column using the following gradient of phase B: 4% to 8% for 10 min, 8% to 20% for 80 min, 20% to 35% for 10 min, 35% to 90% for 5 min, maintained at 95% for 5 min and then back to 4%. The nanoUPLC was coupled online through a nanoESI emitter (10 μm tip; New Objective, Woburn) to a quadrupole Orbitrap mass spectrometer (Q Exactive, Thermo Scientific) using a FlexIon nanospray apparatus (Proxeon). Data was acquired in the DDA mode using the Top12 method [14]. Raw data was imported into TransOmics software (Waters) (also known as Progenesis LC-MS). The software was used for retention time alignment and peak detection of precursor peptides. A master peak list was generated from all MS/MS events and analyzed using Mascot v2.4 (Matrix Sciences). Data was searched against forward and reversed human sequences of UniprotKB version 05_2012 including 125 common laboratory contaminants. Fixed modification was set to carbamidomethylation of cysteines and variable modification was set to oxidation of methionines. Search results were then imported back to TransOmics to annotate identified peaks. Identifications were filtered such that the global false discovery rate was no more than 1%. Differential analysis was conducted by direct comparison of aligned peptide intensities across all samples. Technical replicates were averaged and a Student’s t-Test, after logarithmic transformation, was used to identify significant differences in the biological replicas. The mass spectrometry proteomic data set was deposited in the ProteomeXchange Consortium [15] via the PRIDE partner repository with the dataset identifier PXD001654.

### Western blot analysis

Brain homogenates and CSF samples were electrophoresed on a 10% SDS-polyacrylamide gel and transferred to a nitrocellulose membrane. Blots were incubated with the following primary antibodies: anti-GPNMB (R&D systems, catalog number AF2550), anti-albumin (Dako Cytomation, catalog number A0001), anti-Tau (Cell signaling, catalog number 4019S), anti-mouse P-Tau (Cell signaling, catalog number 9632S), anti-human P-tau (Santa Cruz, catalog number 101815) and anti-AT8 (Innogenetics, catalog number 90206), followed by a horseradish peroxidase-conjugated secondary antibody. Bound antibodies were detected using the SuperSignal West Pico Chemiluminescent substrate (Thermo Scientific).

### Enzyme-Linked Immunosorbent Assay

GPNMB levels were measured in CSF aspirated from type 3 GD patients and in brain tissue from type 2 and 3 GD patients. Brain tissues were lysed in ∼6 volumes of Ripa buffer (150 mM sodium chloride, 1.0% Triton X-100, 0.5% sodium deoxycholate, 0.1% SDS, 50 mM Tris, pH 8.0) supplemented with a protease inhibitor mixture (Sigma-Aldrich). Following homogenization, samples were centrifuged at 4,500*g*_av_ for 5 min at 4°C and the supernatant collected. Protein was quantified using the BCA protein assay reagent (Thermo Scientific). GPNMB levels were quantified using the human GPNMB ELISA kit (R&D systems) according to manufacturer’s instructions.

### GPNMB quantification in mouse CSF and brain

Mice were anesthetized using 100 mg/kg ketamine and 10 mg/kg xylazine, and CSF aspirated from the cisterna magna using a glass needle. Brains were removed and lysed in ∼6 volumes of 150 mM sodium chloride, 1.0% Triton X-100, 0.5% sodium deoxycholate, 0.1% SDS and 50 mM Tris, pH 8.0 supplemented with a protease inhibitor mixture (Sigma-Aldrich). Following homogenization, samples were centrifuged at 4,500xg_av_ for 5 min at 4°C, and the supernatant collected. Protein was quantified using the BCA protein assay reagent (Thermo Scientific). GPNMB was measured using a mouse GPNMB ELISA kit (R&D systems) according to manufacturer’s instructions.

## Results and Discussion

**Fig 1:**
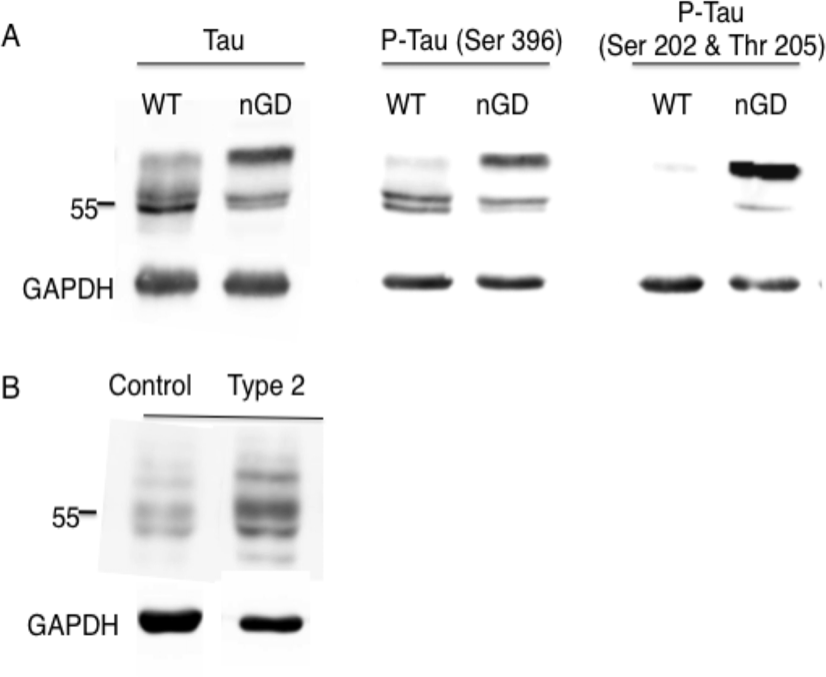
Hyperphosphorylation of Tau in nGD samples. (A) Western blot of Tau and P-Tau (using two different anti-P-Tau antibodies) in brains of 21 day-old Gba^flox/flox^; nestin-Cre mice and (B) P-Tau in the brain of one type 2 GD patient. Results are from a typical experiment repeated 3 times which gave similar results. GAPDH was used as a loading control. A molecular weight marker is shown (Mr = 55 kDa)

Quantitative global proteomic analysis of CSF samples from four type 3 GD patients and 5 age-matched controls was performed. 489 proteins were identified in CSF but only 7 proteins were elevated more than 2-fold (Table 1), and levels of 10 proteins were reduced. Most of the former are involved in the immune response and in lipid metabolism. Of the proteins whose levels were reduced, the most significant reduction was for amyloid βA4 (2.3-fold reduction, p<0.05), a key protein in the pathology of Alzheimer's disease [16]. No amyloid formation could be detected in the brain at the end-stage of Gba^flox/flox^; nestin-Cre mice (not shown), although hyper-phosphorylated tau, a key player that is linked to amyloid formation in Alzheimer's disease [17], was detected in Gba^flox/flox^; nestin-Cre mice (Fig. 1A). Hyper-phosphorylated tau was also detected in a type 2 GD patient (Fig. 1B). Elevated tau was also recently detected in the brain of type 3 GD patient [18].

**Table 1:** Up-regulated proteins in the CSF of type 3 GD patients.

Gene symbol	Protein	Fold change and p value
CHIT1	Chitotriosidase-1	57.7 (<0.01)
GPNMB	Transmembrane glycoprotein NMB	42.3 (<0.01)
SCTSS	Isoform 2 of Cathepsin	6.7 (<0.05)
IGKC	Ig kappa chain V-III region GOL	6.6 (<0.01)
LYZ	Lysozyme C	6.3 (<0.01)
CFD	Complement factor D	3.1 (<0.01)
PLD3	Phospholipase D3	2.4 (<0.01)

CSF from type 3 GD patients and age matched controls (n = 4) was digested with trypsin and subjected to label-free quantitative global proteomic analysis using liquid chromatography and tandem mass spectrometry (LC-MS/MS).

**Fig 2:**
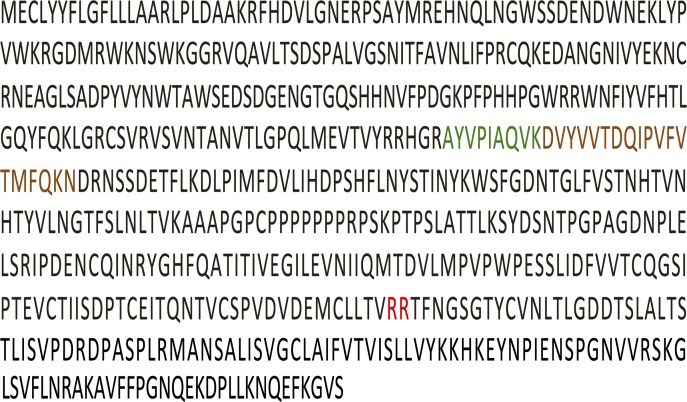
GPNMB peptides identified by LC-MS/MS. The GPNMB sequence is shown (UniProtKB/Swiss-Prot Q14956), with the site of cleavage [20] indicated in red and the two peptides identified by LC-MS/MS indicated in green and brown.

**Fig 3:**
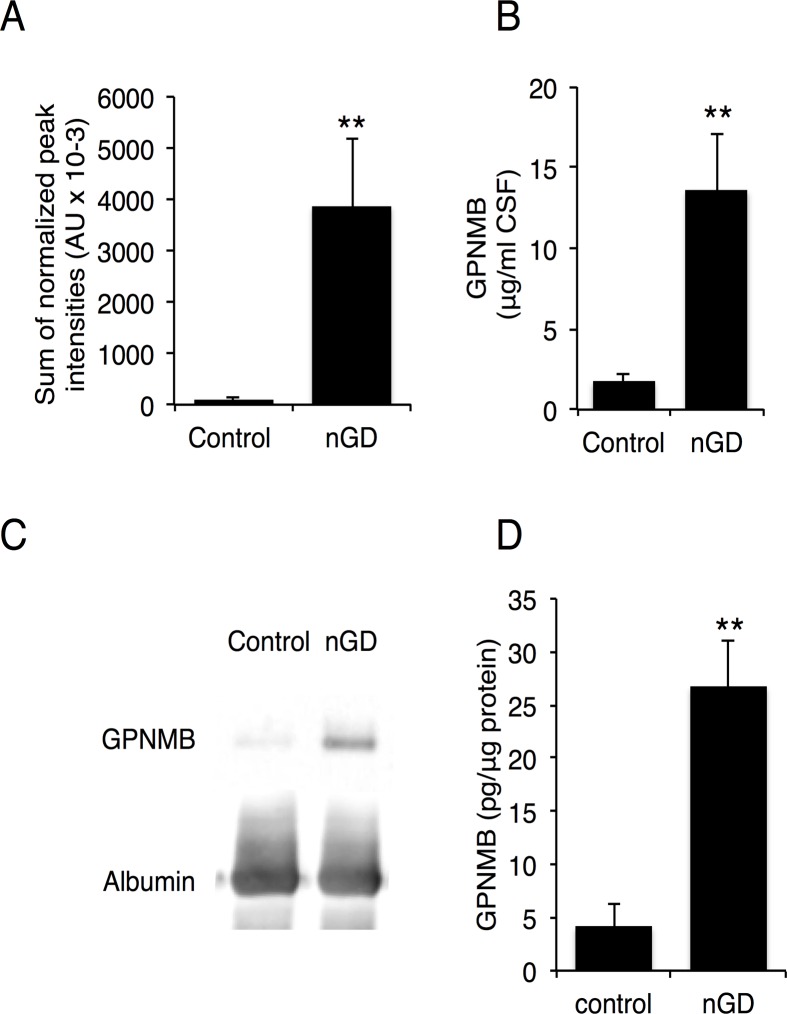
Elevation of GPNMB levels in CSF and brain of nGD patients. (A) Levels of GPNMB determined by LC-MS/MS in CSF of four type 3 GD patients. Results are means ± SEM. ** *p*< 0.01. (B) Levels of GPNMB in CSF of four type 3 GD patients determined by ELISA. Results are means ± SEM (n = 4). ** *p*<0.01. (C) Western blot of GPNMB in CSF of control and a type 3 GD patient (sample designation 4). Results are from a typical experiment repeated 3 times. (D) Levels of GPNMB in nGD brain determined by ELISA(n = 3 for control, n = 6 for nGD (type 2 and type 3 patients). Results are means ± SEM, ** *p*< 0.01

**Fig 4:**
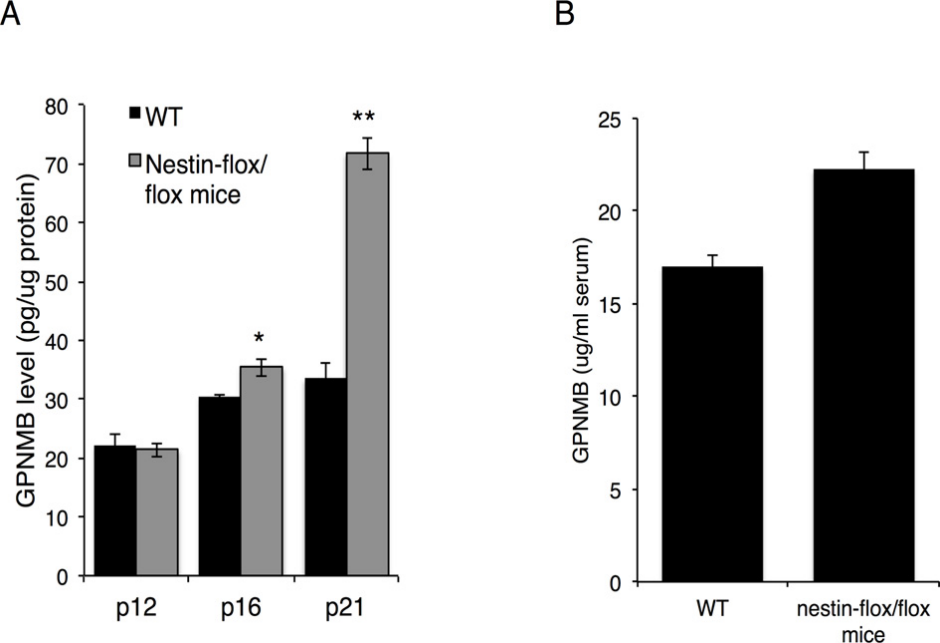
GPNMB levels in brain and serum of Gba^flox/flox^; nestin-Cre mice. Levels of GPNMB in (A) brain (n = 3) at different days post-natal (p) and (B) serum (n = 4,n = 5) of 21-day old Gba^flox/flox^; nestin-Cre mice determined by ELISA. Results are means ± SEM *P < 0.05, **P < 0.01.

Two proteins were noticeably elevated in CSF obtained from type 3 human GD patients, namely chitotriosidase-1 (CHIT1), a known biomarker for GD [19], which was elevated 58-fold, and glycoprotein non-metastatic B (GPNMB) protein, which was elevated 42-fold (Table 1). Two peptides were identified from GPNMB, both located in the non-cytosolic domain [20] (Fig. 2), suggesting that GPNMB is cleaved and secreted into the CSF from the brain. LC-MS/MS results (Fig. 3A) were validated by ELISA (Fig. 3B) and by western blot analysis (Fig. 3C) in CSF and in human brain (Fig. 3D). Thus, three independent techniques corroborated the elevation of GPNMB, suggesting that GPNMB might be a biomarker for following the progression of CNS pathology in nGD patients. Moreover, there was a correlation between GPNMB levels and disease severity, such that higher CSF levels of GPNMB correlated with more severe disease symptoms (assessed by full scale IQ and eye-hand coordination assessed by the Purdue Pegboard Test, Table 2). The elevated levels of GPNMB in the brain of Gba^flox/flox^; nestin-Cre mice, in which GlcCerase deficiency is restricted to neurons and macroglia [11,12], confirms that GPNMB in the CSF does not originate from the periphery but rather directly from the brain (Fig. 4A); however, a small (1.3-fold elevation) of GPNMB was detected in the serum of the Gba^flox/flox^; nestin-Cre mice (Fig. 4B) perhaps suggesting some leakage of the CSF into the serum at the late stage in disease progression at which these analysis were performed. Together, these results suggest that GPNMB levels in the CSF could be used as a biomarker to quantify nGD severity.

**Table 2:** Clinical information and correlation with GPNMB levels in the CSF of type 3 GD patients.

Sample designation	Age (years)	Gender	Genotype	FSIQ*	Purdue Pegboard test	Clinical observations	GPNMB levels (ELISA) (μg/ml)	GPNMB levels (LC-MS/MS) (AUx10^3^ ^#^)
1	16	Female	L444P/L444P	124	-1.71	Eye movement abnormality; mild GD3	6.4	792
2	8	Female	L444P/L444P	74	-2.93	Sensorineural hearing loss	19.5	3,932
3	13	Male	L444P/L444P	45	-3.72	Mental retardation	19.4	4,838
4	15	Male	P122S/P122S	40	-8.73	Progressive myoclonic encephalopathy (Designated as mild GD type 3A with presumed neuronal death)	29.6	5,958

* Full scale IQ

^#^ Arbitrary units

**Fig 5:**
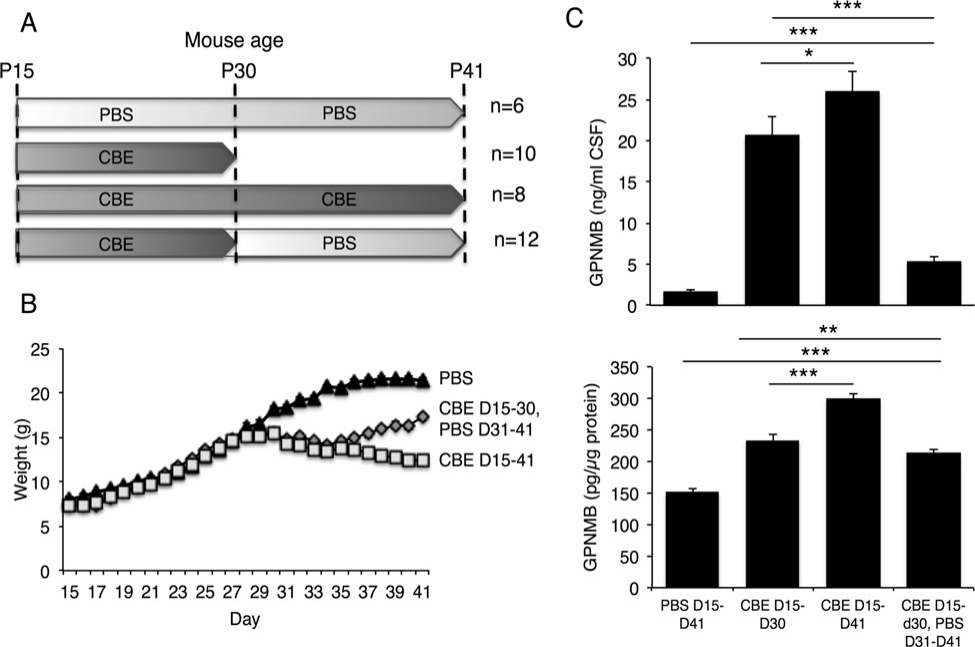
GPNMB levels in CSF and brain of CBE-treated mice. (A) CBE (100 mg/kg, i.p) was injected into 15-day old mice and injections either ceased on day 30 or continued to day 41. When CBE injection was stopped on day 30, mice were injected with phosphate buffered saline (PBS) until the end of the experiment. The number of mice used is shown. (B) Mouse weight. (C) GPNMB levels in the CSF (*upper panel*) and in the brain (*lower panel*) measured by ELISA. Results are means ± SEM. * *p*<0.06, ** *p*<0.01.

To further assess the relationship between CSF levels of GPNMB and disease severity, mice were injected daily, starting on day 15, with relatively high levels (100 mg/kg) of CBE, followed by cessation of CBE injection (Fig. 5A). Mouse weight (which can be used as a simple indicator of disease progression) [12], began to decrease ~10–12 days after beginning CBE injections and continued to decrease until day 41 (Fig. 5B). However, mice in which CBE injections ceased on day 31 began to gain weight (Fig. 5B). GPNMB levels in the CSF correlated with changes in mouse weight and with neurological signs of nGD. Thus, GPNMB levels increased upon injection of CBE from day 15–30 and to a higher extent with injection till day 41 (Fig. 5C). Upon cessation of CBE injection on day 30, a significantly lower level of CSF GPNMB was detected on day 41 than in mice that were continuously treated with CBE until day 41 (Fig. 5C).

To confirm the purity of the mouse CSF samples used in this study, the percent of polynuclear cells in CSF was analyzed by FACS. Polynuclear cells comprised ~0.55% of the cells in the CSF (n = 5), whereas blood samples contained 10% polynuclear cells, indicating the high purity of the CSF samples. Together, these results suggest that if an effective treatment was available for types 2 and 3 GD, analysis of GPNMB levels in the CSF would provide a means to determine treatment efficacy.

In summary, we demonstrate that GPNMB, a transmembrane protein that is expressed in various cell types including melanocytes, osteoclasts, macrophages, neurons and astrocytes [21,22], is significantly elevated in the CSF of nGD patients and that there is a clear correlation between GPNMB levels and disease severity in both human and mouse tissues. Previous studies have shown that GPNMB mRNA levels are elevated in the serum of GD mice [23] and in the spleen of GD patients [24]. Although the purity of the CSF used in the current study excludes the possibility that the GPNMB in the CSF results from non-neuronal tissue, we cannot completely exclude the possibility of perivascular cells originating from the brain in the CSF samples [25]. Elevated levels of GPNMB have also been previously shown in the liver of Niemann Pick type C mice [26] and in some other LSDs, such mucopolysaccharidosis (MPS) VII but not in MPS I or MPS IIIb mice [27], and in the brains of Tay-Sachs and Sandhoff patients [7,28]. Interestingly, GPNMB was also detected in the CSF of amyotrophic lateral sclerosis (ALS) patients [22] suggesting that GPNMB might also be useful as a biomarker to detect deterioration in neurodegenerative diseases in addition to its use in nGD. GPNMB levels are elevated in a number of inflammatory diseases [29–32]. It is secreted from macrophages and acts as a negative regulator of excessive inflammatory responses [21]. Thus, GPNMB levels may be elevated in order to moderate the inflammatory response, such as that which occurs in nGD [4].

## Supporting Information

S1 Dataset (PPTX)

S2 Dataset (XLSX)
