# Supplementary material for: Identification of a Biomarker in Cerebrospinal Fluid for Neuronopathic Forms of Gaucher Disease
Source: PLoS One. 2015 Mar 16;10(3):e0120194. doi: 10.1371/journal.pone.0120194 (PMC4361053; doi:10.1371/journal.pone.0120194)
Supplement: S1 Dataset — (PPTX) [file pone.0120194.s001.pptx]

## Slide 1
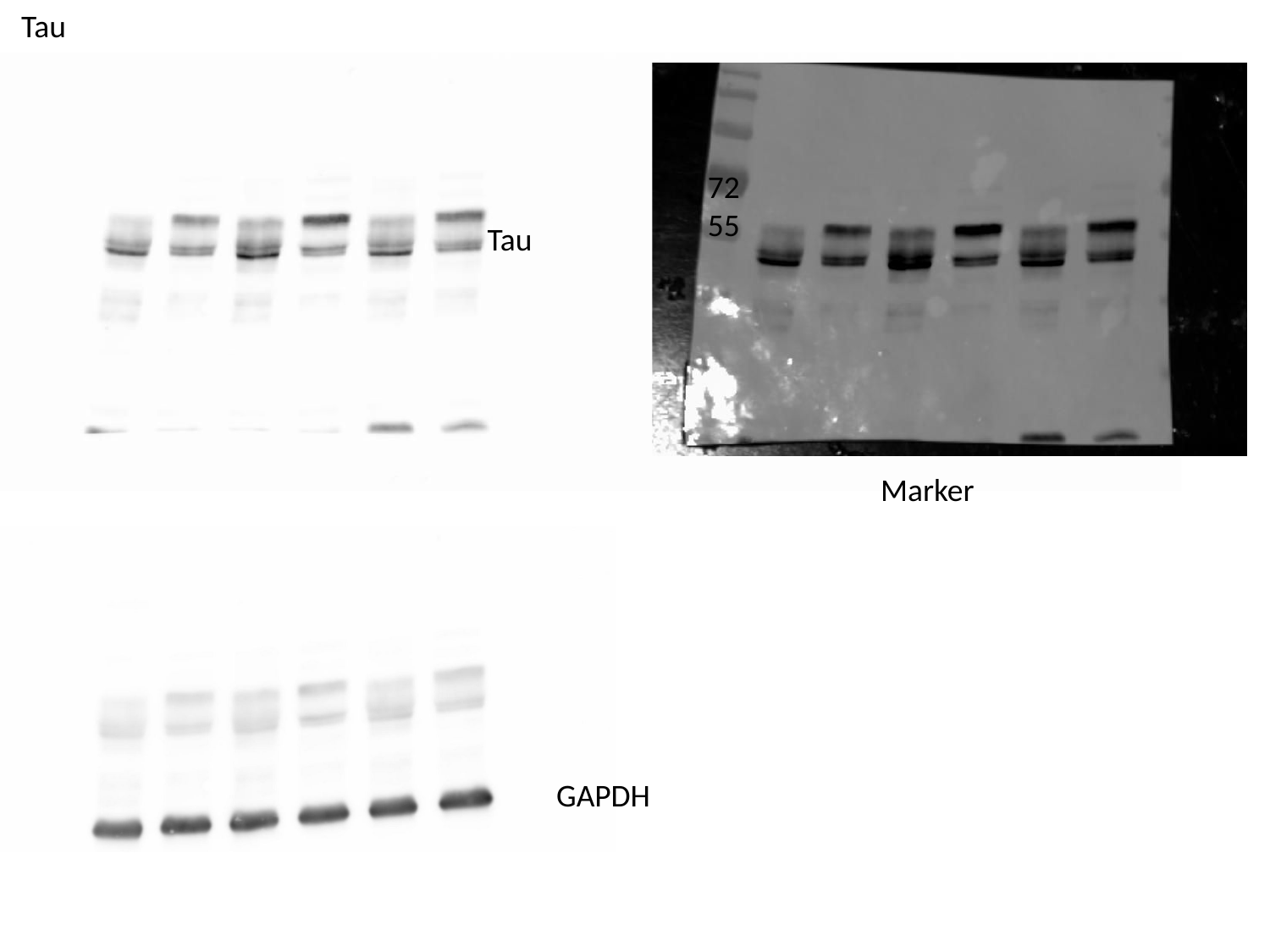

Tau
72
55
Tau
Marker
GAPDH

## Slide 2
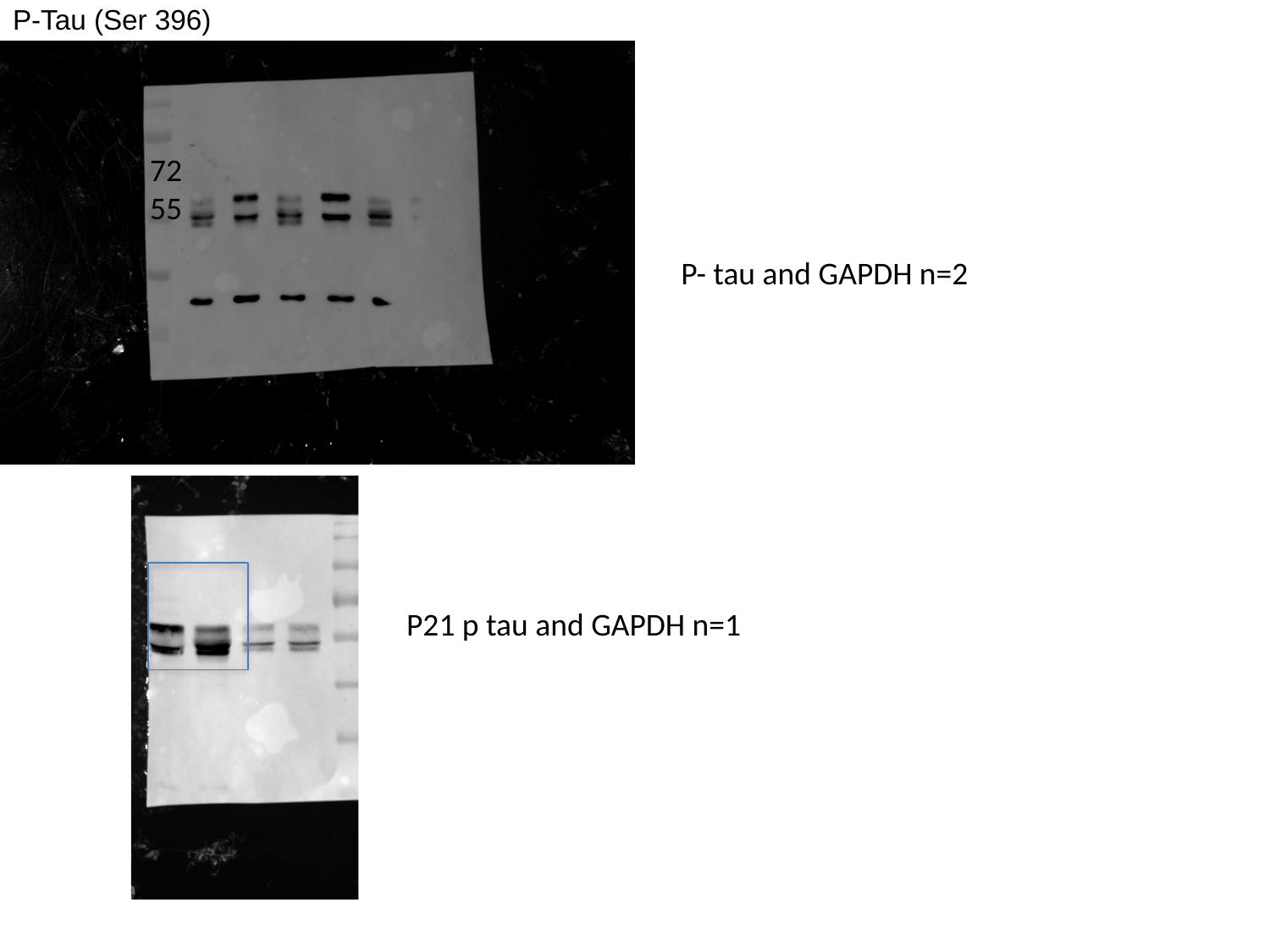

P-Tau (Ser 396)
72
55
 P- tau and GAPDH n=2
P21 p tau and GAPDH n=1

## Slide 3
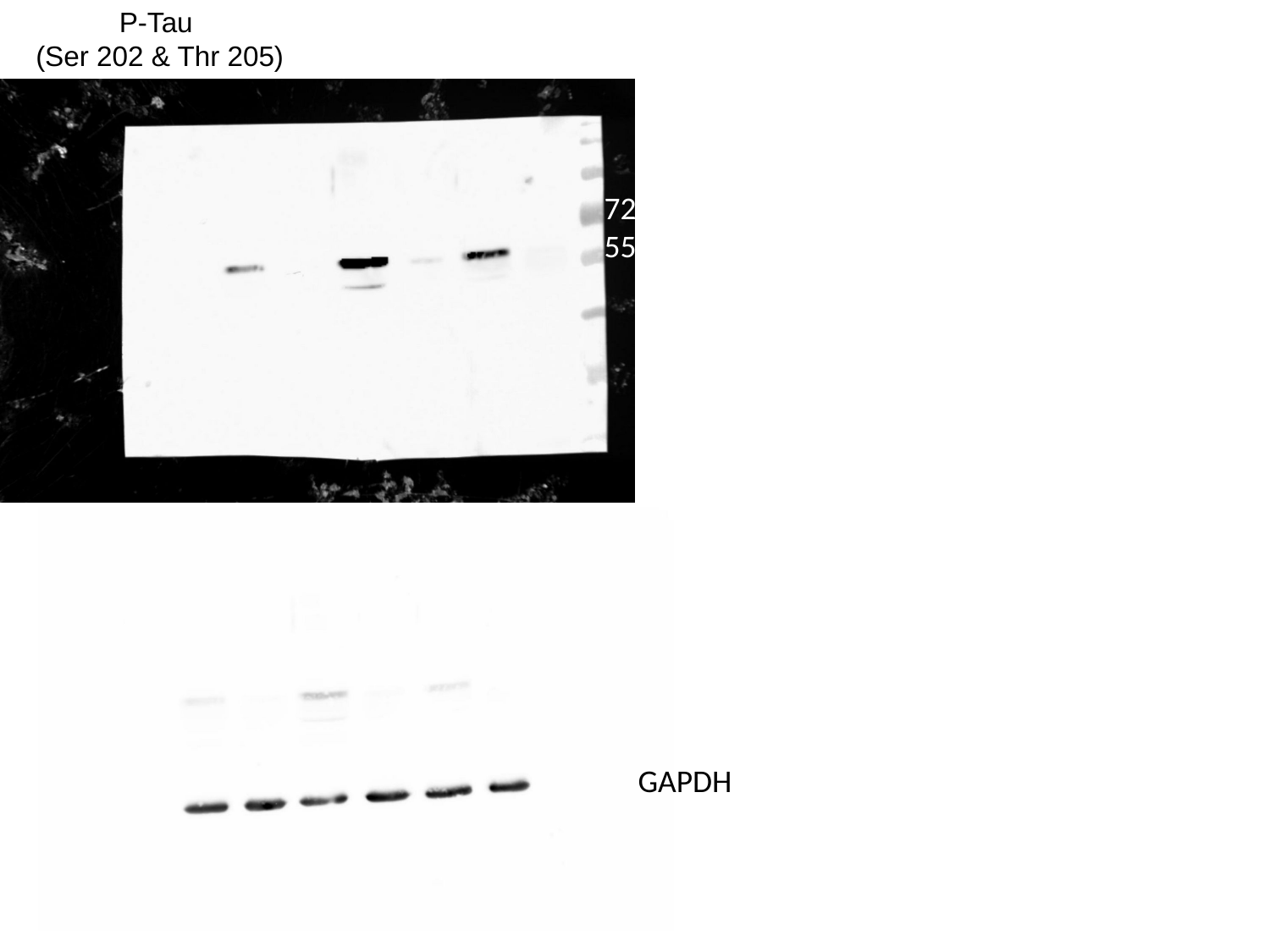

P-Tau
(Ser 202 & Thr 205)
72
55
GAPDH

## Slide 4
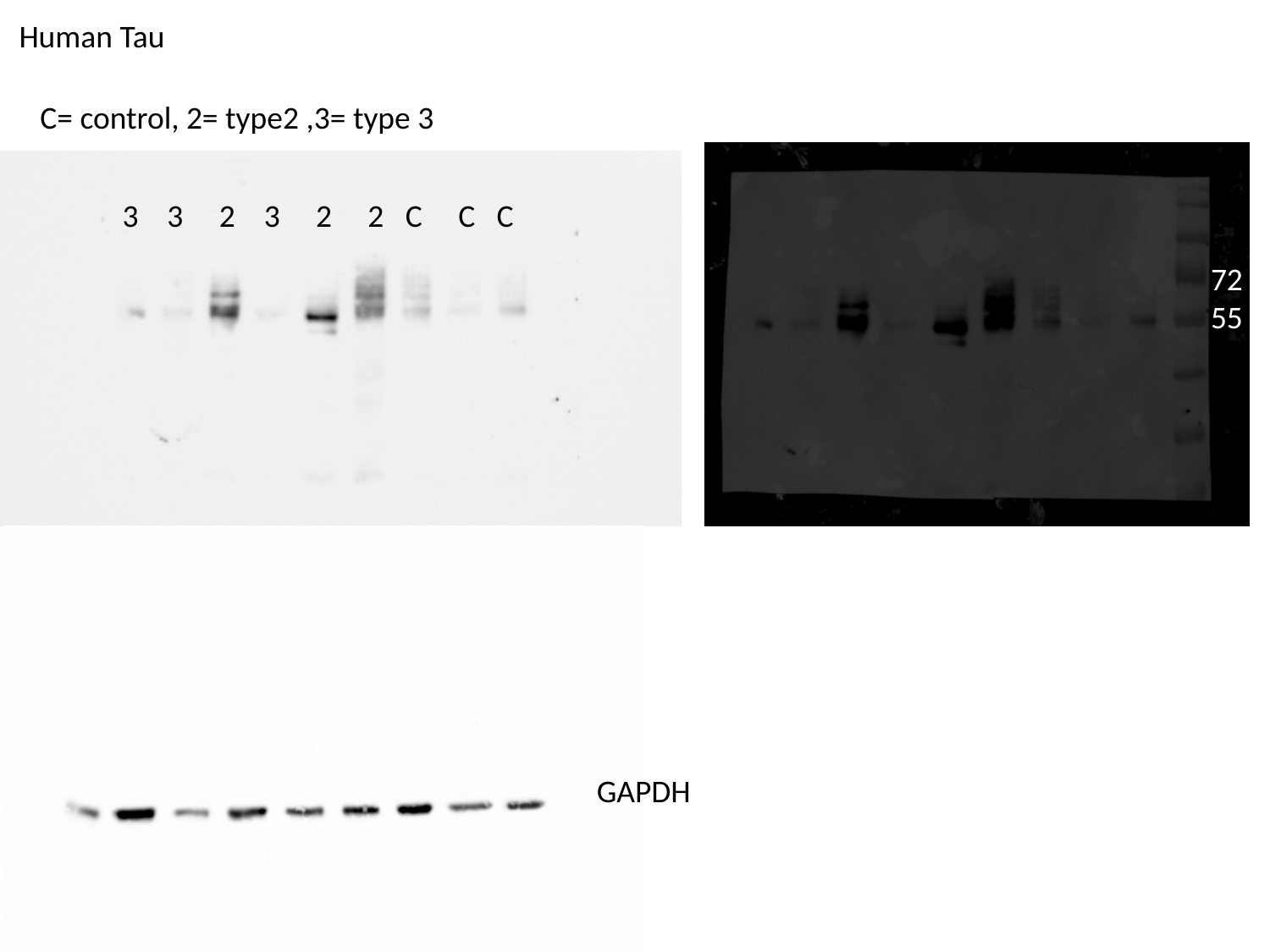

Human Tau
C= control, 2= type2 ,3= type 3
3 3 2 3 2 2 C C C
72
55
GAPDH

## Slide 5
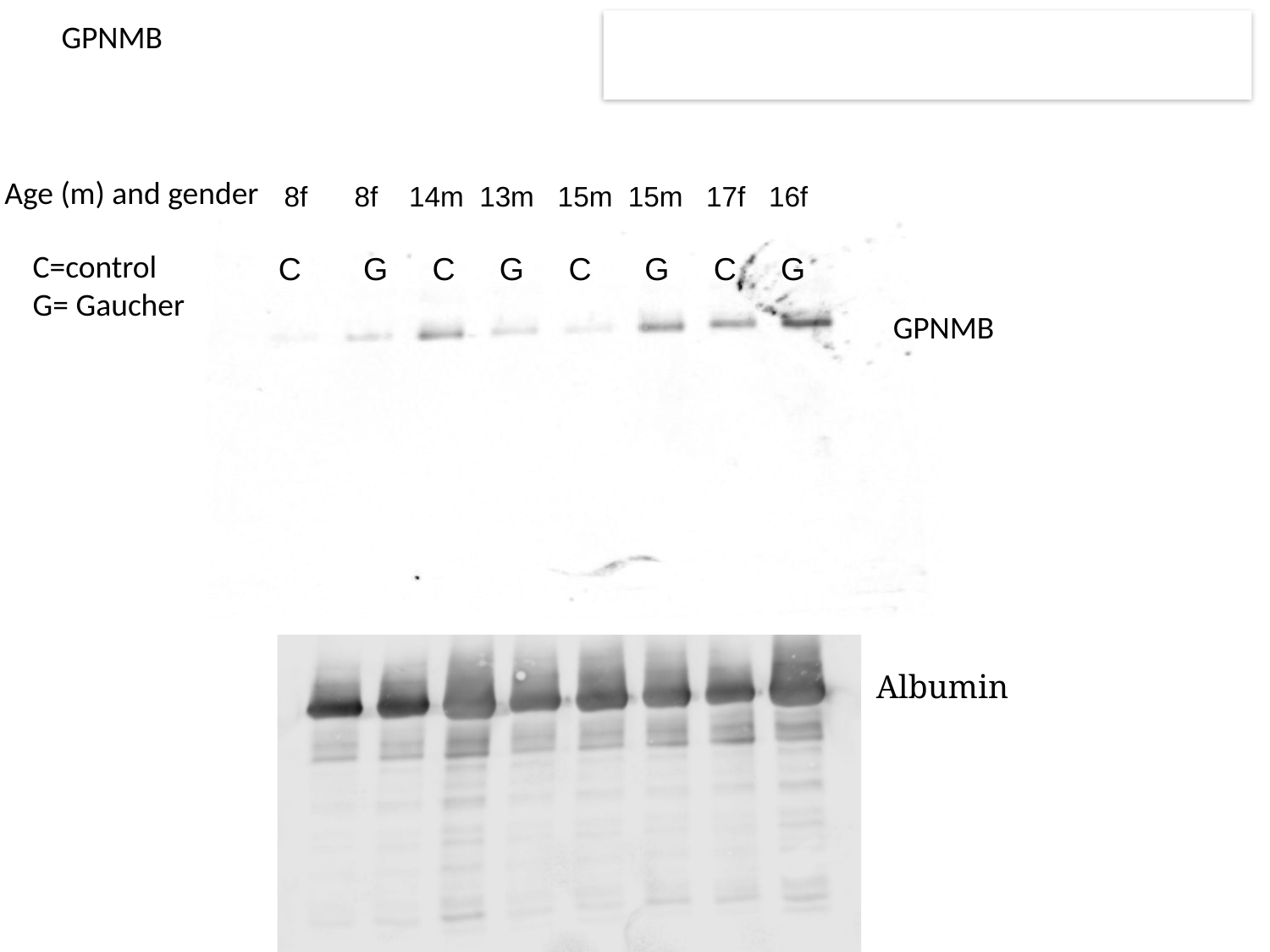

GPNMB
Age (m) and gender
8f 8f 14m 13m 15m 15m 17f 16f
C=control
G= Gaucher
C G C G C G C G
GPNMB
Albumin

## Slide 6
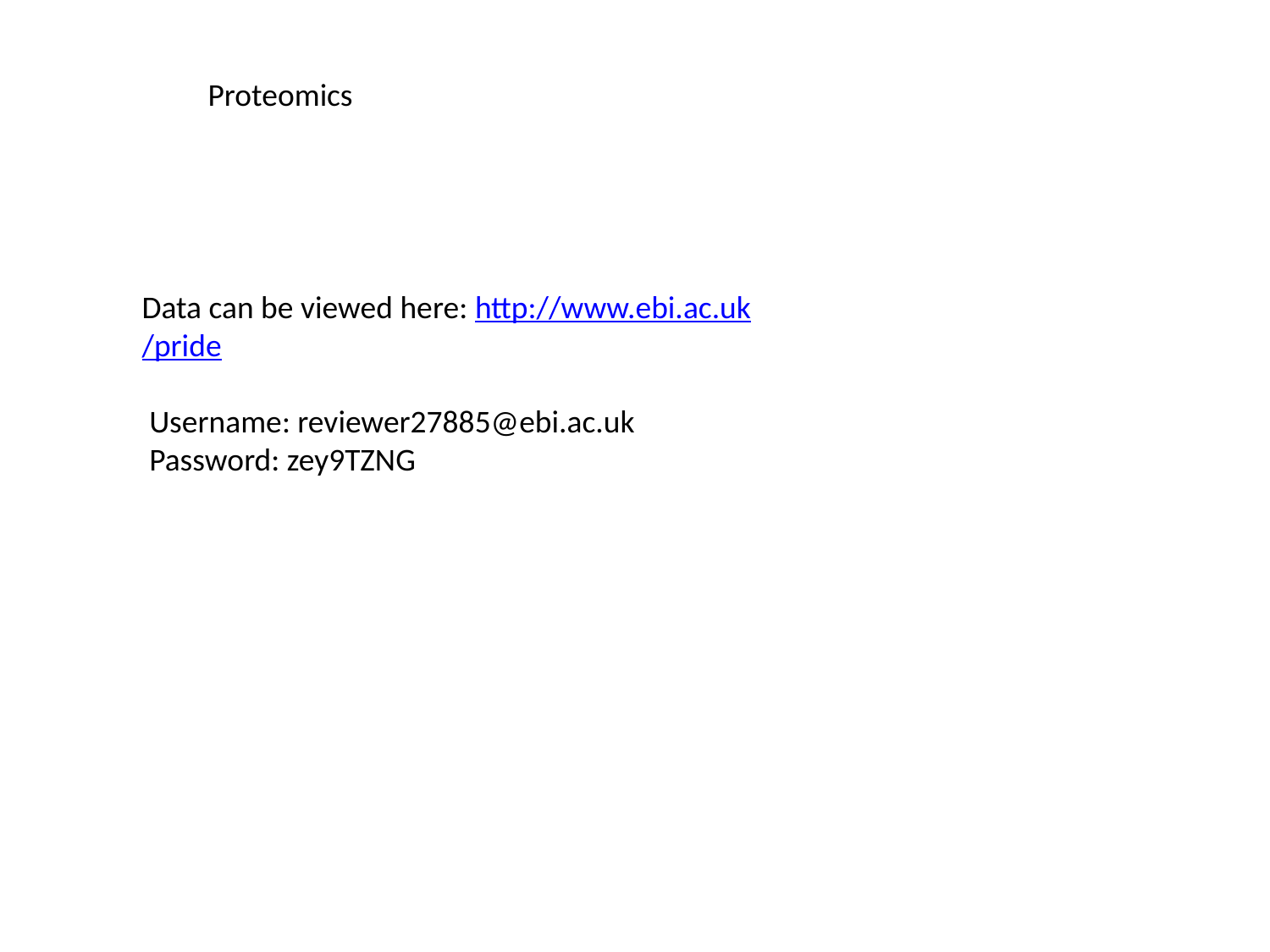

Proteomics
Data can be viewed here: http://www.ebi.ac.uk/pride
 Username: reviewer27885@ebi.ac.uk
 Password: zey9TZNG
